# Dose-Finding Study of a CEA-Targeting Agent, SGM-101, for Intraoperative Fluorescence Imaging of Colorectal Cancer

**DOI:** 10.1245/s10434-020-09069-2

**Published:** 2020-10-09

**Authors:** Kim S. de Valk, Marion M. Deken, Dennis P. Schaap, Ruben P. Meijer, Leonora S. Boogerd, Charlotte E. Hoogstins, Maxime J. van der Valk, Ingrid M. Kamerling, Shadhvi S. Bhairosingh, Bérénice Framery, Denise E. Hilling, Koen C. Peeters, Fabian A. Holman, Miranda Kusters, Harm J. Rutten, Françoise Cailler, Jacobus Burggraaf, Alexander L. Vahrmeijer

**Affiliations:** 1 Centre for Human Drug Research, Leiden, The Netherlands; 2 Department of Surgery, Leiden University Medical Center, Leiden, The Netherlands; 3 Department of Surgery, Catharina Hospital Eindhoven, Eindhoven, The Netherlands; 4 SurgiMab, Montpellier, France; 5 Department of Surgery, Amsterdam University Medical Center, Amsterdam, The Netherlands

## Abstract

**Background:**

Carcinoembryonic antigen is overexpressed in colorectal cancer (CRC), making it an optimal target for fluorescence imaging. A phase I/II study was designed to determine the optimal imaging dose of SGM-101 for intraoperative fluorescence imaging of primary and recurrent CRC.

**Methods:**

Patients were included and received a single dose of SGM-101 at least 24 h before surgery. Patients who received routine anticancer therapy (i.e., radiotherapy or chemotherapy) also were eligible. A dedicated near-infrared imaging system was used for real-time fluorescence imaging during surgery. Safety assessments were performed and SGM-101 efficacy was evaluated per dose level to determine the most optimal imaging dose.

**Results:**

Thirty-seven patients with CRC were included in the analysis. Fluorescence was visible in all primary and recurrent tumors. In seven patients, no fluorescence was seen; all were confirmed as pathological complete responses after neoadjuvant therapy. Two tumors showed false-positive fluorescence. In the 37 patients, a total of 97 lesions were excised. The highest mean intraoperative tumor-to-background ratio (TBR) of 1.9 (*p* = 0.019) was seen in the 10-mg dose. This dose showed a sensitivity of 96%, specificity of 63%, and negative predictive value of 94%. Nine patients (24%) had a surgical plan alteration based on fluorescence, with additional malignant lesions detected in six patients.

**Conclusions:**

The optimal imaging dose was established at 10 mg 4 days before surgery. The results accentuate the potential of SGM-101 and designated a promising base for the multinational phase III study, which enrolled the first patients in June 2019.

**Electronic supplementary material:**

The online version of this article (10.1245/s10434-020-09069-2) contains supplementary material, which is available to authorized users.

Colorectal cancer (CRC) is one of the most commonly diagnosed cancers and fourth-leading cause of cancer death worldwide.^1^ Yet, mortality rates have been stabilizing because of improved screening programs, which have led to earlier detection and prevention through polypectomy.^2^ Additionally, improvements in neoadjuvant therapies have contributed to the subsiding trend in mortality.^3^ Neoadjuvant (chemo-)radiation followed by curative surgery is currently the definitive treatment method for advanced rectal cancer. Neoadjuvant chemoradiation has proven to effectively downstage primary tumors in approximately 20% of patients, where a pathologic complete response (pCR) is realized, with advantageous long-term outcomes.^3^^–^5 Nevertheless, surgery remains the cornerstone treatment of CRC. During surgery, surgeons rely on visual inspection and palpation assessments to differentiate between malignant and benign tissue for resection. Yet, discriminating between these tissues can be challenging after neoadjuvant therapy, due to tumor reduction with subsequent diminished tumor visibility intraoperatively. Also, formation of fibrotic tissue may impair proper assessment of the surgical field. Reduced tumor visibility during surgery can result in unnecessary resections (i.e., benign tissue) or residual malignant tissue (i.e., millimeter lesions or positive resection margins). Therefore, enhancing intraoperative tumor visibility can be a helpful tool for surgeons to aid in achieving R0 resections for better survival.

Near-infrared (NIR) fluorescence imaging is a rapidly evolving technique that allows real-time detection of malignant tissue during surgery.^6^ This technique enhances tumor visibility as it enables the visualization of resection margins and millimeter malignant lesions that are undetectable with the naked eye or preoperative imaging techniques.^7^^–^10 SGM-101 is a tumor-targeting agent consisting of the fluorophore BM-104 covalently bound to the monoclonal antibody that targets carcinoembryonic antigen (CEA), a well-known tumor marker for CRC. CEA is overexpressed in > 90% of CRC cells, with limited expression in normal tissue making it an optimal imaging target for CRC.^11^^,^^12^ Preclinical studies with SGM-101 have shown favorable toxicity profiles in animals, clear delineation of tumors in different colon cancer models, capacity to penetrate and target millimeter-sized tumor nodules and, importantly, make them detectable with fluorescence.^13^^,^^14^ Boogerd et al. published the first efficacy results of SGM-101 in CRC patients.^8^ Results showed SGM-101 up to 10 mg was safe, offered successful detection of primary, recurrent, and metastasized CRC during surgery, and led to an altered treatment plan in one-third of the patients.^8^ Driven by these results, the study was continued to evaluate the safety of higher doses, efficacy of SGM-101 in a larger and more homogenous cohort, and to determine the optimal imaging dose of SGM-101 for the detection of colorectal neoplastic lesions in primary and recurrent CRC patients.

## Methods

### Study Design

**Fig. 1:**
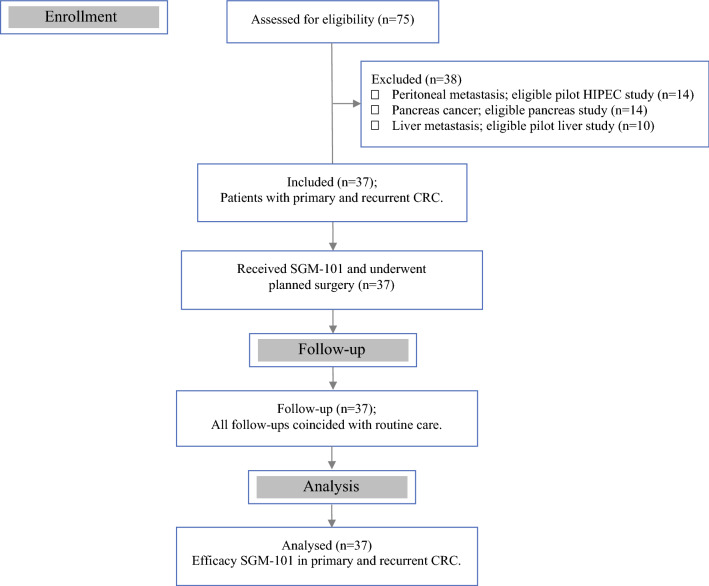
Flow diagram showing the patient inclusion for this study

An ascending dose, exploratory study was performed in 37 patients with primary or recurrent CRC (Fig. 1). Besides safety and efficacy assessments, the study was designed to determine the most optimal dose of SGM-101 for intraoperative fluorescence imaging of colorectal neoplastic lesions. Twenty-one of the 37 patients (9 primary and 12 recurrent CRC) have previously been described in the pilot analysis by Boogerd et al.^8^ The study was a collaborative project performed in the Netherlands between the Centre for Human Drug Research (CHDR) in Leiden and the departments of surgery of Leiden University Medical Center (LUMC) in Leiden and Catharina Hospital Eindhoven (CZE) in Eindhoven. The study was approved by a certified medical ethics review board (Stichting BEBO, Assen, the Netherlands) and was performed in accordance with the laws and regulations on drug research in humans of the Netherlands. The study is registered in ClinicalTrials.gov under identifier NCT02973672.

The study was conducted in five dosing cohorts, where a single dose of SGM-101 (dose range: 5 mg, 7.5 mg, 10 mg, 12.5 mg, or 15 mg per patient) was administered intravenously over 30 min at least 24 h before surgery. Four patients were included in the 5-mg and 7.5-mg dose levels respectively, 19 patients (additional patients included at optimal dose) were included in the 10-mg dose, and five patients were included in the 12.5-mg and 15-mg dose levels, respectively. Patients were admitted to CHDR for SGM-101 administration and were clincially observed for at least 6 h for safety assessments. The dosing-surgery interval of at least 24 h was based on observations in preclinical animal studies and the first-in-human trial (NCT02784028), which showed a sufficient fluorescent signal between 24 and 72 h.^13^ The first five patients in this study were dosed 2 days before surgery. However, the fluorescence results obtained in these patients were not optimal due to strong background fluorescence. This was most likely due to the high concentration of SGM-101 that was still present in the systemic circulation after 2 days.^8^ Consequently, a longer dosing-surgery interval of 4 days was chosen in the subsequent patients. It also was explored whether fluorescence imaging was possible 6 days after SGM-101 administration. Doses were administered in an escalating manner, following review of safety and fluorescence data. In the cohort with the most optimal dose, additional patients were included for further appraisal. Due to the exploratory nature of the study, population size was not based on statistical considerations. Eligible CRC patients were selected during multidisciplinary team meetings and approached at the outpatient clinic by the treating surgeon. All subjects provided written, informed consent before the start of any study-related procedure.

### Surgery

Fluorescence imaging during surgery was performed with the Quest Spectrum Platform (Quest Medical Imaging BV, Middenmeer, The Netherlands), a CE-marked dedicated imaging system optimized for measurements in two NIR channels (700 to 830 nm and 830 to 1100 nm).^15^ Before fluorescence imaging was initiated, visual and/or palpation assessments were done under white light by the surgeon to identify the lesion(s) of interest. Fluorescence imaging was performed before surgical resection and after resection to identify any remaining fluorescence in the surgical field and on the back table. In case fluorescence imaging was warranted throughout other moments, such as during additional resections or inspection, this could be employed. Each lesion was recorded as clinically suspect for malignancy or not and as fluorescent or nonfluorescent. The efficacy of SGM-101 was determined by calculating the tumor-to-background ratio (TBR) of each lesion (intraoperative and back table measurements) and assessing its concordance with tumor histopathology to determine the optimal dose. Intraoperative TBR was defined as the fluorescent signal of the lesion inside the patient before surgical resection. Back table TBR was defined as the fluorescent signal of the lesion after resection on the back table. Back table imaging was implemented to evaluate the resection margins and to determine fluorescence that was possibly not visible intraoperatively. Removal of additional tissue (re-resections and tissue resections elsewhere) or changes in surgical plans due to fluorescence were considered an alteration of initial surgical plan. If removal of a clinically nonsuspect but fluorescent lesion would lead to a larger or complex resection, a frozen section was performed to confirm whether the resection was required. In cases where removal of additional tissue was minor without consequences for the patient, it was the decision of the surgeon.

### Concordance

The resected lesion(s) were sent to the pathology department for assessment according to hospital protocol using standardized Hematoxylin and Eosin (H&E) staining. Of each lesion, tumor status was assessed and correlated with the intraoperative or back table fluorescence to determine concordance. Four different conclusions could be made: a malignant fluorescent lesion was regarded as a true positive (TP); a benign fluorescent lesion was regarded as a false positive (FP); a malignant nonfluorescent lesion was regarded as a false negative (FN); and a benign nonfluorescent lesion was regarded as a true negative (TN). In addition, immunohistochemistry for CEA expression was performed on 4-µm, formalin-fixed, paraffin-embedded sections. To correlate SGM-101 fluorescence with tumor status and CEA expression on a microscopic level, sections were scanned using the Odyssey imager (LI-COR Biosciences, NE).

### Fluorescence and Statistical Assessment

Fluorescence assessments were done by study personnel who also were present during surgery. After surgery, images were viewed and processed using the software Architector Vision Suite version 1.8.3 (Quest innovations, Middenmeer, the Netherlands) and analyzed with ImageJ 1.51j8 (National Institute of Health, MD) to quantify the TBR. The TBR was calculated by drawing a region of interest (ROI) around tumor fluorescence and the directly surrounding background area. The ROI of the tumor was then subtracted from the background. The quotient of the tumor and background signal strength constituted the TBR. This was done for every fluorescent lesion identified. The efficacy analysis was done with SAS software version 9.4 (SAS Institute, Cary, NC).

## Results

From January 2016 to January 2019, a total of 37 patients (23 males and 14 females) with primary (*n* = 16) or recurrent (*n* = 21) CRC were analysed. The majority of patients (30/37 = 81%) received neoadjuvant therapy, consisting of either chemoradiation (*n* = 27), radiation alone (n = 1) or chemotherapy alone (*n* = 2). The rest of the patients (7/37 = 19%) had surgery first. All patients received SGM-101 and underwent surgery according to standard of care. The performed procedures included low anterior resections (*n* = 13), abdominoperineal resections (*n* = 10), sigmoid resections (*n* = 2), recurrence resections (*n* = 8), total exenterations (*n* = 2), hemicolectomy (*n* = 1), and a pancreaticoduodenectomy with colon resection (*n* = 1). Table 1 provides a patient overview, including demographics, dosing-surgery intervals, diagnosis, type of neoadjuvant therapy, type of surgery, and concordance of the primary and recurrent tumors per dose level.

**Table 1:** Patient overview

Demographics			SGM-101 dosing		Diagnosis and treatment			Imaging and concordance to histopathology			
PID	Age (years)	Sex	Dose (mg)	Interval (days)	Diagnosis	Surgical procedure	NAT	Fluorescence	TBR	Histopathology	Concordance
1	67	M	5	2	Primary CRC (rectal)	APR	Chemoradiation	Yes	1.8^a^	Adenocarcinoma	True positive
2	68	F	5	2	Primary CRC (rectal)	APR	Chemoradiation	Yes	1.5	Adenocarcinoma	True positive
3	66	M	5	2	Primary CRC (rectal)	LAR	Chemoradiation	No		Complete response	True negative
4	65	M	5	2	Recurrent CRC (sigmoid)	Lymph node resection	None	Yes	1.4	Mucinous adenocarcinoma	True positive
5	60	M	7.5	2	Recurrent CRC (transverse colon)	Pancreaticoduodenectomy	Chemotherapy	Yes	1.4	Intestinal adenocarcinoma	True positive
6	64	M	7.5	4	Primary CRC (sigmoid)	Sigmoid resection	None	Yes	2.1	Adenocarcinoma	True positive
7	63	M	7.5	4	Primary CRC (rectal)	LAR	Chemoradiation	No		Complete response	True negative
8	79	F	7.5	4	Primary CRC (rectal)	LAR	Chemoradiation	Yes	5.0^a^	Adenocarcinoma	True positive
9	75	F	10	4	Primary CRC (ascending colon)	Hemicolectomy	None	Yes	1.9	Adenocarcinoma	True positive
10	69	M	10	4	Primary CRC (rectal)	LAR	Radiotherapy	Not measured—excluded from analysis			
11	69	F	10	4	Primary CRC (sigmoid)	Sigmoid resection	None	Yes	1.8	Adenocarcinoma	True positive
12	63	M	10	4	Recurrent CRC (sigmoid)	LAR	Chemoradiation	Yes	1.7	Adenocarcinoma	True positive
13	53	M	10	4	Recurrent CRC (rectal)	Resection recurrence	Chemoradiation	Yes	1.9^a^	Adenocarcinoma	True positive
14	56	M	10	4	Recurrent CRC (rectal)	Resection recurrence	Chemoradiation	Yes	1.9	Adenocarcinoma	True positive
15	47	F	10	4	Recurrent CRC (sigmoid)	LAR	Chemoradiation	Yes	1.6^a^	Adenocarcinoma	True positive
16	49	M	10	4	Recurrent CRC (rectal)	Resection recurrence	Chemoradiation	Yes	2.0	Low grade intestinal adenocarcinoma	True positive
17	69	F	10	4	Recurrent CRC (rectal)	APR	Chemoradiation	Yes	2.2^a^	Adenocarcinoma	True positive
18	58	F	10	4	Recurrent CRC (rectal)	APR	Chemoradiation	No		Complete response	True negative
19	62	F	10	4	Recurrent CRC (sigmoid)	Resection recurrence	Chemoradiation	Yes	1.5	Fibrosis; no tumor cells	False positive
20	67	M	10	4	Recurrent CRC (rectal)	Total exenteration	Chemoradiation	Yes	1.7^a^	Sarcomatous differentiated carcinoma	True positive
21	63	M	10	4	Recurrent CRC (rectal)	Resection recurrence	Chemoradiation	Yes	2.4^a^	Adenocarcinoma	True positive
22	71	M	10	4	Recurrent CRC (sigmoid)	APR	None	Yes	1.5^a^	Adenocarcinoma	True positive
23	51	M	10	4	Recurrent CRC (rectal)	Resection recurrence	Chemoradiation	Yes	1.5^a^	Adenocarcinoma	True positive
24	75	M	10	4	Recurrent CRC (ascending colon)	Resection recurrence	None	Yes	1.5	Fibrosis; no tumor cells	False positive
25	43	M	10	4	Recurrent CRC (sigmoid)	LAR	Chemoradiation	No		Complete response	True negative
26	59	M	10	6	Recurrent CRC (sigmoid)	LAR	Chemoradiation	No		Complete response	True negative
27	55	F	10	6	Primary CRC (rectal)	LAR	Chemoradiation	Yes	1.4^a^	Adenocarcinoma	True positive
28	56	F	12.5	3	Recurrent CRC (rectal)	APR	Chemoradiation	Yes	1.8^a^	Adenocarcinoma	True positive
29	44	M	12.5	4	Primary CRC (sigmoid)	LAR	None	Yes	2.0	Adenocarcinoma	True positive
30	63	M	12.5	4	Primary CRC (rectal)	LAR	Chemoradiation	Yes	1.4^a^	Adenocarcinoma	True positive
31	61	F	12.5	4	Primary CRC (rectal)	LAR	Chemoradiation	No		Complete response	True negative
32	58	M	12.5	4	Recurrent CRC (rectal)	APR	Chemoradiation	Yes	1.8^a^	Mucinous adenocarcinoma	True positive
33	75	M	15.0	4	Primary CRC (rectal)	APR	Chemoradiation	Yes	1.1^a^	Adenocarcinoma	True positive
34	58	M	15.0	4	Primary CRC (rectal)	LAR	Chemoradiation	Yes	1.1	Adenocarcinoma	True positive
35	61	F	15.0	4	Recurrent CRC (rectal)	APR	Chemoradiation	Yes	1.9	Adenocarcinoma	True positive
36	57	F	15.0	6	Primary CRC (rectal)	APR	Chemotherapy	No		Complete response	True negative
37	72	F	15.0	6	Recurrent CRC (rectal)	Total exenteration	Chemoradiation	Yes	1.6^a^	Vital tumor cells	True positive

In the diagnosis column, the data in parenthesis indicate the location of the primary tumor

*PID* patient ID, *NAT* neoadjuvant therapy, *TBR* tumor-to-background ratio, *CRC* colorectal cancer, *APR* abdominal perineal resection, *LAR* low anterior resection

^a^Indicates ex vivo TBR, because no in vivo TBR measurement was done or possible

### Safety and Tolerability

All doses up to 15 mg SGM-101 were well tolerated. None of the patients experienced an allergic reaction or event that was considered of clinical importance or led to discontinuation. None of the reported adverse events (AEs) or serious adverse events (SAEs) had a direct causal relationship to SGM-101. A total of 36 post-dose AEs and five SAEs were recorded in 37 patients (Supplementary Table 1). The majority of the AEs were unrelated (*n* = 30) to SGM-101 and considered general postoperative complications. Six AE’s were judged as possible (*n* = 5) or unlikely (*n* = 1) related and included symptoms, such as headache (*n* = 3), abdominal pain (*n* = 1), rash (*n* = 1) and redness of a finger (*n* = 1). The five SAEs (renal injury and paralytic ileus in the 5-mg dose, pyelonefritis in a patient receiving 10 mg, hepatic necrosis most likely due to an infection in the 12.5-mg dose, and cerebral hemorrhage in a patient receiving 15 mg) were all interpreted as unrelated to SGM-101 and related to the surgical procedure or disease. Patients dosed with a higher dose of SGM-101 did not experience more AEs or SAEs.

### Primary and Recurrent Colorectal Tumors

**Fig. 2:**
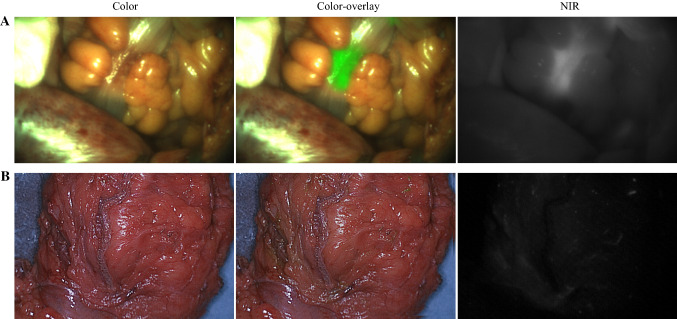
Example of a true positive and a true negative colorectal carcinoma. **A** Intraoperative fluorescence of a palpable colorectal tumor during surgery, with a TBR of 2.0 (true positive). **B** Absence of fluorescence in a tumor, which was confirmed as a pathological complete response by histopathology (true negative). *TBR* tumor-to-background ratio, *NIR* near-infrared

In all primary and residual tumors (*n* = 27), fluorescence was visible during surgery. There were seven patients, all treated with neoadjuvant therapy, where no fluorescence could be detected in the primary (*n* = 4) or recurrent (*n* = 3) tumor. These patients had a pCR, conforming the absence of fluorescence. Figure 2 shows an example of intraoperative fluorescence in a true positive tumor and absence of fluorescence in a true negative case (pCR after neoadjuvant therapy). One tumor was excluded as no fluorescence was measured due to logistic reasons. In two recurrent CRC tumors, fluorescence was detected; however, histopathology showed no malignancy (Table 1). One of these false positives was a suspected recurrent tumor mass near the left iliopsoas muscle, which emitted fluorescence. Histopathology showed extensive necrosis and fibrosis with mucin-producing cells positive for CEA, possibly clarifying the binding of SGM-101.^8^ The second false positive was a suspected recurrent tumor against the retroperitoneum with evident intraoperative and back table fluorescence. Histopathology showed fat tissue with fibrosis and inflammation without malignancy. Additional immunochemistry showed weak CEA expression in epithelial tissue, conceivably explaining SGM-101 binding and fluorescence (Supplementary Fig. 1).

### Total Excised Lesions

In 37 patients, a total of 97 lesions were excised, including the primary and recurrent tumors (Supplementary Table 2). Of the 97 excised lesions, 49 lesions were malignant and 48 lesions benign. Of the 49 malignant lesions, 47 lesions were true positive (96%). Unfortunately, not all true-positive lesions could be identified intraoperatively due to anatomic positioning but were fluorescent on the back table after excision. Merely 20 of the 47 true-positive lesions (43%) were visible intraoperatively. Two malignant lesions were false negative (no intraoperative or back table fluorescence). One false negative contained a conglomerate of three metastasized lymph nodes with adenocarcinoma (10-mg dose) and CEA expression in immunohistochemistry. The second false negative was a frozen-section biopsy of a resected tumor with mucinous adenocarcinoma (12.5-mg dose). This biopsy was performed to evaluate the resection margin, because the surgeon had doubts on the radicality, despite the absence of fluorescence. Conversely, immunohistochemistry of the biopsy showed no CEA expression, which complements the absence of fluorescence. Of the 48 benign lesions, 26 lesions were true negatives (54%). There were 22 false-positive lesions, which included lymph nodes, reproductive organs (i.e., ovaries and vesicula seminalis) and frozen-section biopsies of resection planes and tissue around the sacrum and pelvic floor. These biopsies were taken to determine whether an additional resection was needed.

### Additional Lesions and Alteration in Surgical Plan

Initial surgical plan alterations due to SGM-101 fluorescence occurred in 12 of the 37 patients (Table 3). In nine patients, the alteration was warranted (24%). In seven of these patients, additional tissue was removed after initial tumor resection. A total of eight additional malignant lesions were identified in six patients, which were otherwise left behind. These lesions were not visible with white light and clinically unsuspect for malignancy. They were only visible with fluorencence with a mean intraoperative TBR of 1.8 (SD 0.06). Lesions were generally detected after initial tumor resection when imaging of the surgical field was performed to assess remaining fluorescence. Two patients had downstaging of the surgical plan due to absence of fluorescence, confirmed benign with frozen sections. Hence, the resections in these two patients were assessed as radical which resulted in tissue salvaging around the lateral piriformis in one patient and omitting intraoperative radiotherapy (IORT) on the sciatic nerve in the second patient. In the remaining three patients, the additional tissue removed were false positive. The false-positive tissue removed was minor and did not have consequences for the patient. Details on the surgical plan alterations are provided in Table 3.

### Performance and Optimal Dose

**Fig. 3:**
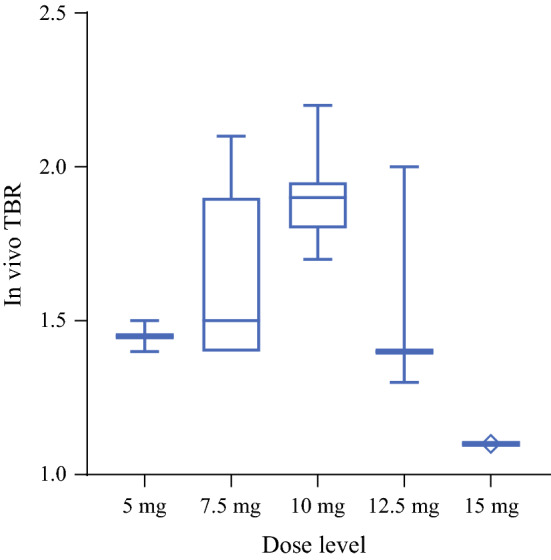
Intraoperative tumor-to-background ratio (TBR) per dose level. Median and range of the intraoperative TBRs. *Note*: The dosing-surgery interval time varies within the different dose levels

The mean intraoperative TBR of the true positive lesions for the 5-mg, 7.5-mg, 10-mg, 12.5-mg, and 15-mg dose levels are 1.5 (SD 0.07), 1.6 (SD 0.30), 1.9 (SD 0.15), 1.6 (SD 0.38), and 1.1 (SD 0.00), respectively (Fig. 3; *p* = 0.019 one-way ANOVA). Fluorescence did not improve at higher doses. Surgical observations and TBR measurements showed that 10 mg of SGM-101 with a dosing-surgery interval of 4 days was the most optimal, resulting in additional patient inclusions in this dosing regimen. It should be taken into consideration that not all patients in the study had the same dosing-surgery interval. Due to relatively small patient numbers in each dose, the TBR was calculated per dose and not split per dosing-surgery interval. Nonetheless, the majority of patients (27/37 patients) had a dosing-surgery interval of 4 days. The dose 10 mg revealed a sensitivity of 96%, a specificity of 63%, and a negative predictive value of 94% (Table 2). Supplementary Figure 2 provides an overview of the back table TBRs.

**Table 2:** Efficacy of SGM-101 per dose level

Dose (mg)	TP	FP	FN	TN	Sensitivity	Specificity	PPV	NPV	Accuracy	False-positive rate
5	3	1	0	1	3/3 (100%)	1/2 (50%)	3/4 (75%)	1/1 (100%)	4/5 (80%)	1/2 (50%)
7.5	7	3	0	2	7/7 (100%)	2/5 (40%	7/10 (70%)	2/2 (100%)	9/12 (75%)	3/5 (60%)
10	23	10	1	17	23/24 (96%)	17/27 (63%)	23/33 (70%)	17/18 (94%)	40/51 (78%)	10/27 (37%)
12.5	7	3	1	3	7/8 (88%)	3/6 (50%)	7/10 (70%)	3/4 (75%)	10/14 (71%)	3/6 (50%)
15	7	5	0	3	7/7 (100%)	3/8 (38%)	7/12 (58%)	3/3 (100%)	10/15 (67%)	5/8 (63%)

The sensitivity, specificity, positive predictive value, negative predictive value and accuracy of SGM-101 was calculated per dose level. The concordance of all 97 resected lesions are included in the analysis

*TP* true positive, *FP* false positive, *FN* false negative, *TN* true negative, *PPV* positive predictive value, *NPV* negative predictive value

**Table 3:** Initial surgical plan alterations due to SGM-101 fluorescence

Treatment	PID	Diagnosis	Procedure	Alteration	Description lesion	Conclusion
5 mg of SGM-101	3	Primary rectal cancer	LAR	Additional resection	Clinically non suspect fluorescent lesion on the bladder wall; resected per decision of the surgeon	Histopathology = benign; bladder dysplasia False positive
10 mg of SGM-101	12	Recurrent sigmoid cancer	LAR	Additional resection	Clinically non suspect fluorescent lesion near the left ureter; resected per decision of the surgeon	Histopathology = malignant True positive: additional lesion
	14	Recurrent rectal cancer	APR	Additional resection	Clinically non suspect fluorescent lesion proximal of recurrence; resected per decision of the surgeon	Histopathology = malignant True positive: additional lesion
	15	Recurrent sigmoid cancer	LAR	Additional resection	Clinically non suspect fluorescent lesion on lateral pelvic wall; resected per decision of the surgeon	Histopathology = malignant True positive: additional lesion
	17	Recurrent rectal cancer	APR	Additional resection	Remaining fluorescence in surgical field (clinically non suspect); two re-resections performed	Histopathology = malignant (2) True positive (2): additional lesions
	21	Recurrent rectal cancer	Resection recurrence	Additional resection	(1) Remaining fluorescence visible in surgical field (clinically non suspect); re-resection performed (2) Clinically non suspect fluorescent lesion on lateral pelvic wall; resected per decision of the surgeon	Histopathology = malignant (2) True positive (2): additional lesions
	22	Recurrent sigmoid cancer	APR	Additional resection	Remaining fluorescence visible in surgical field (clinically non suspect); re-resection performed	Histopathology = benign False positive
	23	Recurrent rectal cancer	Resection recurrence	Additional resection	Remaining fluorescence visible in surgical field (clinically nonsuspect); re-resection performed	Histopathology = benign False positive
12.5 mg of SGM-101	32	Recurrent rectal cancer	APR	Additional resection	Clinically nonsuspect fluorescent lesion on lateral pelvic wall; resected per decision of the surgeon	Histopathology = malignant True positive: additional lesion
15 mg of SGM-101	34	Primary rectal cancer	LAR	Downstaging treatment	No remaining fluorescence visible in surgical field after resection. Initial surgical plan included a more extensive resection; however, due to negative fluorescence (confirmed benign with FS), the surgeon decided to salvage tissue around the lateral piriformis	Histopathology = benign True negative
	35	Recurrent rectal cancer	APR	Additional resection	Remaining fluorescence visible in surgical field (clinically suspect); re-resection performed	Histopathology = malignant True positive^a^
	36	Primary rectal cancer	APR	Downstaging treatment	No remaining fluorescence visible in surgical field or specimen after resection. The surgeon assessed the resection as radical, which was confirmed with FS (surgical field and specimen). The surgeon decided to spare the patient the planned IORT, because this would have been on the sciatic nerve, giving morbidity	Histopathology = benign True negative

*CRC* colorectal carcinoma, *LAR* low anterior resection, *APR* abdominal perineal resection, *FS* frozen section, *IORT* intraoperative radiotherapy

^a^Lesion was clinically suspect for malignancy, therefore not judged as an additional lesion

## Discussion

This dose finding study in primary and recurrent CRC patients showed that doses up to 15 mg SGM-101 are safe, but that 10 mg of SGM-101 was the most optimal in exposing CRC and, more importantly, in detecting neoplastic lesions that were invisible with white light.

A strong benefit of SGM-101 in the study was the identification of additional malignant lesions that were clinically not suspected for malignancy or invisible with the naked eye but only detectable under fluorescence. In six patients, SGM-101 was of additional value, because it led to the removal of additional malignant tissue that was otherwise left behind. This bares great potential as current preoperative imaging modalities are known to have a detection threshold of approximately 1 cm.^16^ Due to this limited resolution, lesions less than 1 cm can be left undetected, causing undesirable uncertainty in oncologic staging and treatment.^8^^,^^16^

A noteworthy result is that SGM-101 has a high NPV (94%) and sensitivity (96%) with the dose of 10 mg, making it a promising tool for the management of CRC. This opens the possibility for clinical decision making based on fluorescence, with the prospect of salvaging tissue. It also accentuates SGM-101 can play an important role in watch-and-wait (W&W) strategies after neoadjuvant therapy, when combined with NIR endoscopy.^17^ W&W was implemented to avoid morbidity by preventing unneccesary surgery in patients with a clinical complete response (cCR) after neoadjuvant therapy.^18^^,^^19^ In recent years, W&W has been gaining popularity as different studies have shown it is a good alternative to major surgery with little oncological risk.^17^^,^^20^^,^^21^ The identification of cCR is best accomplished with a combination of rectal exam, endoscopy, and high-resoluton imaging [i.e., magnetic resolution imaging (MRI)].^3^^,^^22^^,^^23^ Because local regrowths occur in 25% of all patients and are almost exclusively (97%) situated within the bowel wall, endoscopy is the preferred screening technique.^3^ The use of NIR endoscopy can offer a safe and effective method to monitor tumor regrowth in real-time. Malignant tissue, especially small regrowths that are negligible on current imaging modalities, can be probed efficiently using NIR light and perhaps improve the early detection in W&W.^16^ In this current report, all patients with a pCR after neoadjuvant therapy (*n* = 7) had no fluorescence on the remaining scar tissue, substantiating the value of SGM-101 in complete response cases.

Intraoperative and back table TBR measurements were obtained to evaluate the performance of SGM-101 and concordance to histopathology at each dose level. Intraoperative TBR is considered leading, as the decision to resect tissue is done during surgery. On the other hand, decision making on intraoperative fluorescence has its drawbacks for deep-seated tumors, precluding proper imaging due to the anatomic position. The use of NIR light allows detection of structures or tissues up to 1 cm in depth. Yet, this penetration depth has shown to be insufficient for mesorectal fat around deeply seated rectal tumors, because this is too thick.^8^ Therefore, back table imaging should also be taken into account, especially for the recognition of resection margins in possible R1 resections. Another profit of back table imaging can be to improve the guidance of IORT in cases of tight resection margins. The majority of patients underwent surgery in CZE, which is a tertiary referral center for IORT. Indications for IORT have been described before and is applied in patients with close involved margins, such as in locally advanced and recurrent rectal cancer.^24^ This also has been incorporated in Dutch Oncology Guidelines, suggesting that in aforementioned cases IORT may have added value for local control.

Because not all malignant lesions could be identified intraoperatively, mainly due to anatomic positioning and depth, it was decided concordance assessment should be based on both intraoperative and back table fluorescence. However, several considerations must be kept in mind with this approach. Combining intraoperative and back table fluorescence diminishes the chance that a lesion is labelled false negative, as it is not detected during the surgical procedure itself. Yet, back table imaging is necessary to provide essential information on resection margins and supplementary fluorescence assessment in cases of deep-seated tumors, which restrict optimal intraoperative imaging, substantiating the combined approach.

A shortcoming of the study is the unequal amount of patients in each dose level, resulting in a suboptimal comparison. Likewise, the dosing-surgery time interval varies between patients, possibly hampering a fair comparison of intraoperative TBRs per dose. Yet, all patients with an intraoperative TBR in the 10-mg dose had a dosing-surgery interval of 4 days. Furthermore, it is clear that a higher dose of 12.5 mg and 15 mg did not result in higher TBRs, which can conceivably be explained by the higher intensity of background fluorescence.

In the study, false-positive lesions were found in 22 of the 97 excised lesions. False-positive lesions could be explained by CEA positivity found in histiocytes within lymph nodes, in fibrotic and chronic inflamed tissue, as well as the presence of mucin producing cells, which express CEA, explaining SGM-101 uptake.^8^ Hypotheses for the lesions without CEA expression may be the use of NIR light around the 700-nm wavelength. It is known that this wavelength has higher tissue autofluorescence when compared to 800 nm. Collagen-rich structures, calcifications or the sacral bone could have likely triggered fluorescence during surgery, due to its autofluorescence properties. Perhaps, conjugation of the anti-CEA monoclonal antibody to a NIR 800-nm dye could limit this problem. Studies have indicated that longer-wavelength dyes have increased penetration depth, enhanced sensitivity for small tumor deposits detection, and generally a lower background signal.^25^ Several preclinical studies have been performed with anti-CEA conjugated to a NIR 800 nm dye, such as IRDye-800CW, which revealed successful tumor specificity and distribution of the tracer.^26^^,^^27^ Besides wavelength limitations, the enhanced permeability and retention (EPR) effect also could play a part in the high false positive rate, as hypervascularization and compromised lymphatic drainage can result in non-specific accumulation of SGM-101 in tissue.^28^^,^^29^ It is however conspicuous that a majority of the false positives were found in the reproductive organs (i.e., ovaries and vesicula seminalis). Yet, why these organs emit fluorescence without containing tumor cells cannot clearly be explained and should be inspected in ensuing studies.

This study confirms that SGM-101 is a safe tumor-targeted fluorescence imaging agent for CRC. The dose 10 mg with a dosing-surgery interval of 4 days is the most favorable regimen for implementation and further exploration in CRC patients. The results emphasize the potential of SGM-101 and have led to a follow-up multinational phase III study to provide supportive data for changing the standard of care in CRC patients. A multicenter, randomized, controlled phase III study (NCT03659448) is currently ongoing evaluating SGM-101 in a larger homogenous population to assess the efficacy in terms of clinical benefits in additional lesion detection and its influence in radical resection (R0) rates, which should ultimately result in improved local control and overall survival in CRC patients.

## Electronic supplementary material

Below is the link to the electronic supplementary material.

Supplementary material 1 (PDF 316 kb)
