# Supplementary material for: Dose-Finding Study of a CEA-Targeting Agent, SGM-101, for Intraoperative Fluorescence Imaging of Colorectal Cancer
Source: Ann Surg Oncol. 2020 Oct 9;28(3):1832–44. doi: 10.1245/s10434-020-09069-2 (PMC7892528; doi:10.1245/s10434-020-09069-2)

Supplementary Materials of the Manuscript:

"Dose-finding study of a CEA-targeting agent, SGM-101, for intraoperative fluorescence imaging of colorectal carcinoma"

de Valk et al

Supplementary Table 1: Overview of (serious) adverse events

Cohort	System organ class	Symptom	Severity	SAE	Relationship SGM-101	Occurrence
**5.0 mg SGM-101**						
Patient 1	Skin and subcutaneous tissue disorders	Scar pain	Mild	No	Unrelated	Single occasion
	Gastrointestinal disorders	Ileus paralytic	Mild	No	Unrelated	Single occasion
	General disorders and administration site conditions	Pyrexia	Mild	No	Unrelated	Single occasion
	Gastrointestinal disorders	Proctalgia	Mild	No	Unrelated	Single occasion
Patient 2	Gastrointestinal disorders	Nausea	Mild	No	Unrelated	Single occasion
Patient 3	Metabolism and nutrition disorders	Dehydration	Mild	No	Unrelated	Single occasion
Patient 4	Infections and infestations	Urinary tract infection	Mild	No	Unrelated	Single occasion
	Injury, poisoning and procedural complications	Scratch	Mild	No	Unrelated	Single occasion
	Gastrointestinal disorders	Nausea	Mild	No	Unrelated	Intermittent
	Vascular disorders	Scrotal hematoma	Mild	No	Unrelated	Single occasion
	Gastrointestinal disorders	Ileus paralytic	Moderate	Yes	Unrelated	Single occasion
	Injury, poisoning and procedural complications	Renal injury	Moderate	Yes	Unrelated	Single occasion
**7.5 mg SGM-101**						
Patient 6	Skin and subcutaneous tissue disorders	Rash	Mild	No	Possible	Intermittent
	Vascular disorders	Systemic inflammatory response syndrome	Moderate	No	Unrelated	Single occasion
	Vascular disorders	Orthostatic hypotension	Mild	No	Unrelated	Intermittent
Patient 7	Gastrointestinal disorders	Nausea	Mild	No	Unrelated	Single occasion
Patient 5	General disorders and administration site conditions	Flank pain	Mild	No	Unrelated	Single occasion
**10.0 mg SGM-101**						
Patient 9	Nervous system disorders	Headache	Mild	No	Possible	Single occasion
	Injury, poisoning and procedural complications	Phlebitis	Mild	No	Unrelated	Intermittent
	Gastrointestinal disorders	Nausea	Mild	No	Unrelated	Single occasion
Patient 10	Gastrointestinal disorders	Abdominal pain	mild	No	Possible	Intermittent
	Musculoskeletal and connective tissue disorders	Muscle tightness	Mild	No	Unrelated	Single occasion
Patient 11	Musculoskeletal and connective tissue disorders	Redness finger	Mild	No	Unlikely	Single occasion
	Cardiac disorders	Atrial fibrillation	Mild	No	Unrelated	Single occasion
	Skin and subcutaneous tissue disorders	Decubitus ulcer	Mild	No	Unrelated	Single occasion
	Infections and infestations	Wound infection	Mild	No	Unrelated	Single occasion
Patient 27	Infections and infestations	Pneumonia	Mild	No	Unrelated	Single occasion
	Nervous system disorders	Neuropathy peripheral	Mild	No	Unrelated	Single occasion
Patient 13	Infections and infestations	Urinary tract infection	Mild	No	Unrelated	Single occasion
Patient 14	Renal and urinary disorders	Pyelonephritis	Moderate	Yes	Unrelated	Single occasion
Patient 16	Nervous system disorders	Headache	Mild	No	Possible	Single occasion
Patient 20	Nervous system disorders	Headache	Mild	No	Possible	Single occasion
Patient 23	Nervous system disorders	Peripheral sensorimotor neuropathy	Mild	No	Unrelated	Single occasion
Patient 25	Nervous system disorders	Neuropathy peripheral	Mild	No	Unrelated	Persistent
**12.5 mg SGM-101**						
Patient 29	Hepatobiliary disorders	Hepatic necrosis	Moderate	Yes	Unrelated	Single occasion
Patient 28	Infections and infestations	Abdominal abscess	Mild	No	Unrelated	Single occasion
**15.0 mg SGM-101**						
Patient 33	Infections and infestations	Pneumonia	Moderate	No	Unrelated	Single occasion
	Injury, poisoning and procedural complications	Gastroparesis postoperative	Mild	No	Unrelated	Single occasion
Patient 36	Nervous system disorders	Cerebral hemorrhage	Severe	Yes	Unlikely	Persistent
Patient 37	Injury, poisoning and procedural complications	Postoperative ileus	Moderate	No	Unrelated	Single occasion
	Infections and infestations	Postoperative abscess	Moderate	No	Unrelated	Single occasion

Supplementary Figure 1: Recurrent tumour against peritoneum (false positive). Figure A shows the intraoperative images, figure B shows the back table images and figure C shows the CEA staining and fluorescence correlation with the Odyssey on microscopic level. The presence of low and weak CEA expression in the lesion plausibly explains the (false positive) fluorescence during surgery.

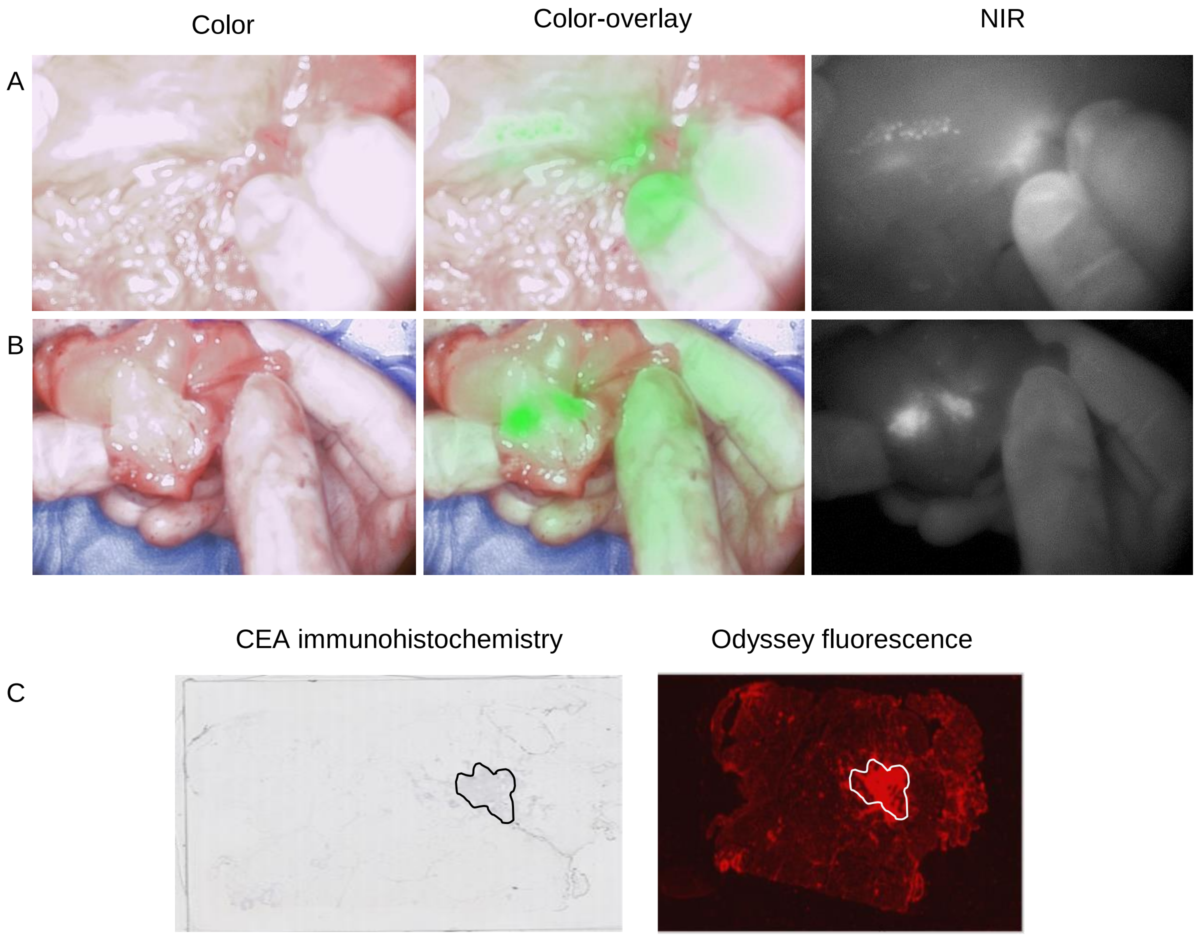

Supplementary Table 2: Overview of excised lesions

Treatment	PID	Resected lesion	Clinically suspect	Fluorescent	TBR in vivo	TBR ex vivo	Pathology	Diagnosis	Conclusion
5 mg SGM-101	1	Tumor rectum	yes	yes	!	1.8	Malignant	Adenocarcinoma	True positive
	2	Tumor rectum	yes	yes	1,5		Malignant	Adenocarcinoma	True positive
	3	Tumor rectum	no	no			Benign	No resttumor: complete response	True negative
		Re-resection bladder wall	no	yes	1,6		Benign	No tumor cells	False positive
	4	Tumor	yes	yes	1,4		Malignant	Mucinous adenocarcinoma	True positive
7.5 mg SGM-101	5	Tumor	yes	yes	1,4		Malignant	Intestinal adenocarcinoma	True positive
		Biopt resection plane	no	yes	1,3		Benign	No tumor cells	False positive
		Biopt resection plane	no	yes	1,3		Benign	No tumor cells	False positive
		Omental lesion	no	no			Benign	Sinus histiocytosis	True negative
		Lymph node	yes	yes	!	1.8	Benign	Sinus histocytosis	False positive
	6	Tumor sigmoid	yes	yes	2,1		Malignant	Adenocarcinoma	True positive
		Lesion liver segment 2/3	yes	yes	1,5		Malignant	Adenocarcinoma	True positive
		Lesion liver 4B	yes	yes	1,7		Malignant	Adenocarcinoma	True positive
		Lesion liver 6	yes	yes	1,4		Malignant	Adenocarcinoma	True positive
	7	Tumor rectum	no	no			Benign	No resttumor: complete response	True negative
	8	Tumor rectum	yes	yes	!	5.0	Malignant	Adenocarcinoma	True positive
		Lymph node	yes	yes	!	1.9	Malignant	Adenocarcinoma	True positive
10 mg SGM-101	9	Tumor colon	yes	yes	1,9		Malignant	Adenocarcinoma	True positive
		Ingrowth omental	yes	yes	2,2		Malignant	Adenocarcinoma	True positive
		Lymph node	yes	no			Malignant	Adenocarcinoma	False negative
	10	Biopt lesion peritoneum	no	yes	1,7		Benign	Ink spot; no tumor cells	False positive
	11	Tumor sigmoid	yes	yes	1,8		Malignant	Adenocarcinoma	True positive
	12	Recurrence anastomosis	yes	yes	1,7		Malignant	Adenocarcinoma	True positive
		Lesion around ureter (LN)	no	yes	1,9		Malignant	Adenocarcinoma	True positive
		Lesion piriformis	no	no			Benign	No tumor cells	True negative
		Lesion left ureter	yes	yes	1,6		Benign	Collagen tissue	False positive
	13	Prostate, recurrence spot	yes	yes	!	1.9	Malignant	Adenocarcinoma	True positive
		Lesion adhesion small bowel	yes	no			Benign	Fibrotic collagen tissue	True negative
	14	Lesion anastomosis, recurrence	yes	yes	1,9		Malignant	Intestinal adenocarcinoma	True positive
		Lesion peritoneum small pelvis	yes	no			Benign	No tumor cells	True negative
		Second lesion	no	yes	!	2.1	Malignant	Intestinal adenocarcinoma	True positive
	15	Tumor	yes	yes	!	1.6	Malignant	Adenocarcinoma	True positive
		LN	yes	no			Benign	No tumor cells	True negative
		Lesion lateral pelvic wall	no	yes	!	1.4	Malignant	Adenocarcinoma	True positive
		Lesion small bowel	yes	yes	1,4		Benign	No tumor cells	False positive
	16	Tumor rectum	yes	yes	2,0		Malignant	Low grade intestinale adenocarcinoma	True positive
	17	Tumor	yes	yes	!	2.2	Malignant	Adenocarcinoma	True positive
		LN	no	yes	!	2.2	Benign	No tumor cells	False positive
		LN AMI	no	no			Benign	No tumor cells	True negative
		Re-excision caudal left	no	yes	1,8		Malignant	Adenocarcinoma	True positive
		Re-excision cranial right	no	yes	1,8		Malignant	Adenocarcinoma	True positive
	18	Tumor rectum	no	no			Benign	No resttumor: complete response	True negative
		Tumor ingrowth vagina	no	no			Benign	No resttumor	True negative
	19	Tumor recurrence spot	yes	yes	1,5		Benign	Tissue with fibrosis	False positive
	20	Sacrum, recurrence spot	yes	yes	!	1.7	Malignant	Sarcomatous differentiated carcinoma	True positive
		Resection plane prostate	no	no			Benign	No tumor cells	True negative
		Tissue between bladder + sacrum	yes	yes	!	1.8	Malignant	Sarcomatous differentiated carcinoma	True positive
	21	Tumor 1	yes	yes	!	3.0	Malignant	Adenocarcinoma	True positive
		Tumor 2	yes	yes	!	1.8	Malignant	Adenocarcinoma	True positive
		LN	no	no			Benign	No tumor cells	True negative
		Lesion lateral pelvic wall	no	yes	!	1.6	Malignant	Adenocarcinoma	True positive
		Re-excision	no	yes	!	1.5	Malignant	Limited (border) adenocarcinoma	True positive
	22	Tumor rectum	yes	yes	!	1.5	Malignant	Adenocarcinoma	True positive
		Re-excision dorsolateral right	no	yes	!	1.4	Benign	Fat and fibrotic connective tissue, bloodvessels	False positive
		Re-ecxcision presacral	yes	yes	!	1.2	Benign	Fat and fibrotic connective tissue, bloodvessels	False positive
	23	Tumor rectum	yes	yes	!	1.5	Malignant	Adenocarcinoma	True positive
		Re-excision tissue	no	yes	!	1.3	Benign	Fat and connective tissue, nerve tissue	False positive
	24	Recurrence retroperitoneum	yes	yes	1,5	1,6	Benign	Fat tissue with fibrosis, macrofages, calcifications	False positive
	25	Tumor rectum	yes	no			Benign	No resttumor: complete response	True negative
	26	Tumor colon	yes	no			Benign	No resttumor: complete response	True negative
		Lesion anastomosis	no	no			Benign	No tumor cells	True negative
		Biopt presacral left	no	no			Benign	No tumor cells	True negative
		Biopt presacral left	no	no			Benign	No tumor cells	True negative
		Biopt presacral	no	no			Benign	No tumor cells	True negative
	27	Tumor rectum	yes	yes	!	1.4	Malignant	Adenocarcinoma	True positive
		Biopt presacral	no	yes	!	1.2	Benign	Fat and connective tissue	False positive
		Biopt presacral left	no	no			Benign	No tumor cells	True negative
		Biopt presacral right	no	no			Benign	No tumor cells	True negative
12.5 mg SGM-101	28	Tumor rectum	yes	yes	!	1.8	Malignant	Adenocarcinoma	True positive
		Vasa ovarica	no	yes	!	1.3	Benign	Ovary stroma, connective tissue, bloodvessels	False positive
	29	Tumor colon	yes	yes	2,0	1.5	Malignant	Adenocarcinoma	True positive
		Lesion liver	yes	yes	1,3		Malignant	Adenocarcinoma	True positive
	30	Tumor rectum	yes	yes	!	1.4	Malignant	Adenocarcinoma	True positive
		Lymph node	yes	yes	1,4	1,5	Malignant	Adenocarcinoma	True positive
		Left vesicula seminalis	no	yes	1,3	1,8	Benign	Vesicula seminalis	False positive
	31	Tumor rectum	yes	no			Benign	No resttumor: complete response	True negative
		Left ovarium	no	yes	1,2	1,5	Benign	Ovary	False positive
	32	Tumor rectum	yes	yes	!	1.8	Malignant	Mucineus adenocarcinoma	True positive
		Lesion lateral pelvic wall	no	yes	!	1.6	Malignant	Mucineus adenocarcinoma	True positive
		Re-excision S4	no	no			Benign	Fibrosis, fat tissue, connective tissue	True negative
		Re-excision S3	no	no			Benign	Fibrosis, fat tissue, connective tissue	True negative
		Biopt tumorwall left side	yes	no			Malignant	Mucineus adenocarcinoma	False negative
15 mg SGM-101	33	Tumor rectum	yes	yes	!	1.1	Malignant	Adenocarcinoma	True positive
		Lesion (possible LN)	yes	yes	!	1.5	Benign	Vesicula seminalis	False positive
	34	Tumor rectum	yes	yes	1,1	1,3	Malignant	Adenocarcinoma	True positive
		Biopt dorsolateral right	no	no			Benign	No tumor cells	True negative
		Biopt lateral pelvis wall	yes	yes	1,5		Benign	Connective tissue	False positive
		Biopt left vesicula seminalis	no	no			Benign	No tumor cells	True negative
	35	Tumor rectum	yes	yes	!	1.9	Malignant	Adenocarcinoma	True positive
		Re-resection pelvic floor	yes	yes	!	1.5	Malignant	Adenocarcinoma	True positive
	36	Tumor rectum	yes	no			Benign	No resttumor: complete response	True negative
		lymph node packet 1	yes	yes	!	1.4	Malignant	Macrometastasis	True positive
		lymph node packet 2	yes	yes	!	1.5	Malignant	Micrometastasis	True positive
		Denonvillien	no	yes	!	1.2	Benign	Mucine deposit; no tumor cells	False positive
	37	Tumor rectum	yes	yes	!	1.6	Malignant	Vital tumor cells	True positive
		Biopt pelvic floor	no	yes	1,8		Benign	Connective tissue	False positive
		Biopt lateral pelvic wall	yes	yes	2,0	1,8	Benign	Connective tissue	False positive

PID: patient ID; TBR: tumor-to-background ratio
! no in vivo fluorescence measured

Supplementary Figure 2: Ex vivo (back table) tumor-to-background ratio (TBR) per dose level. The figure displays the median and range of the ex vivo TBRs. The mean ex vivo TBRs for the 5 mg, 7.5 mg, 10 mg, 12.5 mg and 15 mg dose levels are 1.8, 3.5, 1.8, 1.6 and 1.5, respectively. Note: the dosing-surgery interval time varies within the different dose levels.

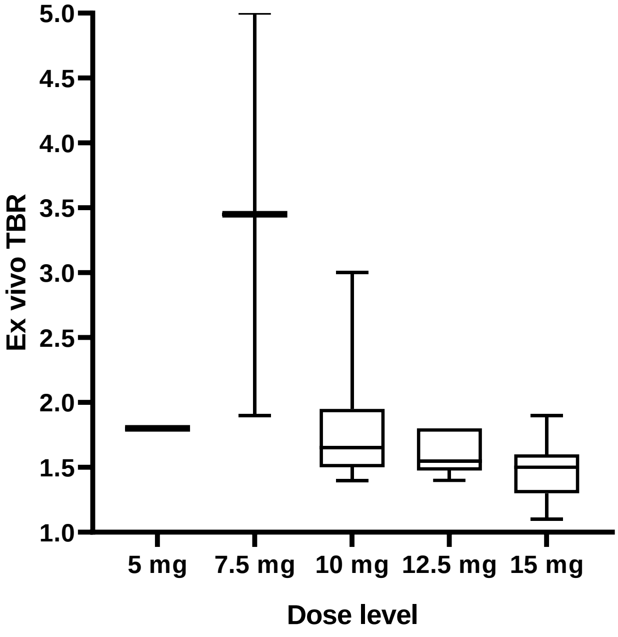

Supplement: Supplementary file 1 — Supplementary material 1 (PDF 316 kb) [file 10434_2020_9069_MOESM1_ESM.pdf]
